# Mendelian randomization analysis of vitamin D in the secondary prevention of hypertensive-diabetic subjects: role of facilitating blood pressure control

**DOI:** 10.1186/s12263-022-00704-z

**Published:** 2022-01-29

**Authors:** Yap-Hang Chan, C. Mary Schooling, Jie V. Zhao, Shiu-Lun Au Yeung, Jo Jo Hai, G. Neil Thomas, Kar-Keung Cheng, Chao-Qiang Jiang, Yuen-Kwun Wong, Ka-Wing Au, Clara S. Tang, Chloe Y. Y. Cheung, Aimin Xu, Pak-Chung Sham, Tai-Hing Lam, Karen Siu-Ling Lam, Hung-Fat Tse

**Affiliations:** 1 Division of Cardiology, Queen Mary Hospital, The University of Hong Kong, Hong Kong, China; 2 School of Public Health, The University of Hong Kong, Hong Kong, SAR China; 3 Department of Medicine, Shenzhen Hong Kong University Hospital, Shenzhen, China; 4 Department of Public Health and Epidemiology, University of Birmingham, Birmingham, UK; 5 Guangzhou No. 12 Hospital, Guangzhou, People’s Republic of China; 6 Department of Psychiatry and Centre for Genomic Sciences, University of Hong Kong, Hong Kong, China; 7 Division of Endocrinology, Queen Mary Hospital, The University of Hong Kong, Hong Kong, China; 8 Hong Kong-Guangdong Joint Laboratory on Stem Cell and Regenerative Medicine, The University of Hong Kong, Hong Kong, China; 9 Shenzhen Institutes of Research and Innovation, The University of Hong Kong, Hong Kong, SAR China

**Keywords:** Vitamin D, Exome Chip, Mendelian randomization, Type 2 diabetes, Hypertension, Secondary prevention, Chinese

## Abstract

**Background:**

Vitamin D (Vit-D) promotes vascular repair and its deficiency is closely linked to the development of type 2 diabetes mellitus (T2DM) and hypertension. Whether genetially predicted vitamin D status (serological 25-hydroxyvitamin D [25(OH)D]) confers secondary protection against cardiovascular diseases (CVD) among high-risk hypertensive-diabetic subjects was unknown.

**Methods:**

This is a prospective, individual-data, two-sample Mendelian randomization study. We interrogated 12 prior GWAS-detected SNPs of comprehensive Vit-D mechanistic pathways using high-throughput exome chip analyses in a derivation subcohort (*n* = 1460) and constructed a genetic risk score (GRS) (*rs2060793*, *rs4588*, *rs7041*; F-statistic = 32, *P* < 0.001) for causal inference of comprehensive CVD hard clinical endpoints in an independent sample of hypertensive subjects (*n* = 3746) with prevailing co-morbid T2DM (79%) and serological 25(OH)D deficiency [< 20 ng/mL] 45%.

**Results:**

After 55.6 ± 28.9 months, 561 (15%) combined CVD events including myocardial infarction, unstable angina, ischemic stroke, congestive heart failure, peripheral vascular disease, and cardiovascular death had occurred. Kaplan-Meier analysis showed that genetically predicted reduced vitamin D status was associated with reduced event-free survival from combined CVD events (log-rank = 13.5, *P* = 0.001). Multivariate-adjusted per-allele increase in GRS predicted reduced combined CVD events (HR = 0.90 [0.84 to 0.96], *P* = 0.002). Mendelian randomization indicates that increased Vit-D exposure, leveraged through each 1 ng/mL genetically instrumented rise of serum Vit-D, protects against combined CVD events (Wald’s estimate: OR = 0.86 [95%CI 0.75 to 0.95]), and myocardial infarction (OR = 0.76 [95%CI 0.60 to 0.90]). Furthermore, genetically predicted increase in Vit-D status ameliorates risk of deviation from achieving guideline-directed hypertension control (JNC-8: systolic target < 150 mmHg) (OR = 0.89 [95%CI 0.80 to 0.96]).

**Conclusions:**

Genetically predicted increase in Vit-D status [25(OH)D] may confer secondary protection against incident combined CVD events and myocardial infarction in a hypertensive-diabetic population where serological 25(OH)D deficiency is common, through facilitating blood pressure control.

**Supplementary Information:**

The online version contains supplementary material available at 10.1186/s12263-022-00704-z.

## Introduction

Latest Mendelian randomization studies showed that deficiency of vitamin D (Vit-D) may play a causal role in the development of both type 2 diabetes mellitus (T2DM) [1–4] and hypertension [5, 6]. These observations sparkle interests to look for a unifying mechanistic connection. Indeed, T2DM and hypertension frequently co-exist and share intertwined etiology [7–11]. Cardiovascular disease (CVD) represents a major disease burden consequential to uncontrolled hypertension and T2DM. Detrimental effects of hypertension and T2DM exhibit a synergistic effect [10, 11]. Nevertheless, the upstream etiological origin of hypertension and T2DM remained incompletely understood.

Extensive studies, including large randomized trials [12, 13], showed that Vit-D was unlikely to play a key role in the primary prevention of CVD. However, prior studies had predominantly focused on healthy subjects with normal baseline serum 25-hydroxyvitamin D [(25(OH)D] concentrations [12, 13]. On the other hand, a protective effect of 25(OH)D against CVD events was noted in high-risk groups, such as diabetic patients [14]. Indeed, increased 25(OH)D was shown in experimental studies to promote vascular regeneration and repair [15]. Its role in the secondary protection of subjects with established CVD or at increased risk was rarely explored.

To test the hypothesis that Vit-D may confer secondary CVD protective effects in the high-risk hypertensive-diabetic population, we performed a prospective Mendelian randomization study to investigate any secondary protective effects of serum 25(OH)D on incident CVD endpoints in a cohort of hypertensive subjects with prevailing type 2 diabetes. We further tested a second hypothesis that genetically predicted Vit-D alters CVD outcomes in these hypertensive-diabetic subjects through modulating blood pressure and/or glycemic control.

## Research design and methods

### Study subjects

This study was performed based on the existing platform of the University of Hong Kong Theme-Based Research Scheme Cohort (HKU-TRS), as detailed elsewhere [16]. Briefly, the HKU-TRS recruited a total of 6048 Southern Chinese subjects, who underwent exome chip-genotyping with individual-level quality control. Subjects were prospectively followed up for incident CVD nonfatal and fatal events. Written informed consent was obtained for all subjects. The study has been approved by the Institutional Review Board, Hong Kong West, Hospital Authority, and adhered to the Declaration of Helsinki.

In the current study, the HKU-TRS cohort sample was methodologically structured and analyzed using a framework for two-sample Mendelian randomization (Supplementary Fig. 1).

#### Derivation subcohort sample

A total of 1460 subjects (mean age: 56.2 ± 11.4 years, 53.8% male) without hypertension at recruitment constituted a *Derivation Subcohort* for deriving the genetic instrument for Vit-D pathways for Mendelian randomization. All included subjects had serum 25(OH)D measurements and were genotyped for Vit-D mechanistic pathway genetic variants. Their clinical and biochemical characteristics are presented in Table 1.

**Table 1:** Derivation and hypertensive-diabetic subcohort sample characteristics, stratified by incident combined cardiovascular (CV) endpoints

	Derivation Subcohort	Hypertensive-Diabetic Subcohort			
	*(i) N = 1460*	*Nil incident event* *(ii) N = 3185*	*Incident CV endpoints(iii) N = 561*		
**Male [*n*** **(%)]**	786 (53.8%)	1993 (62.6%)	354 (63.1%)	< 0.001*	0.81
**Age (years)**	56.2 ± 11.4	64.7 ± 11.5	71.8 ± 10.4	< 0.001*	< 0.001*
**Ever smoking [*n*** **(%)]**	504 (34.5%)	1167 (36.6%)	239 (42.6%)	0.001*	0.007*
**Body mass index (kgm**^**-2**^**)**	24.0 ± 3.5	25.9 ± 4.1	25.9 ± 4.4	< 0.001*	0.89
**Diabetes mellitus [*n*** **(%)]**	473 (32.4%)	2485 (78.0%)	469 (83.6%)	< 0.001*	0.003*
**Systolic blood pressure (mmHg)**	120.0 ± 14.8	139.3 ± 19.4	141.4 ± 21.9	< 0.001*	0.031*
**Diastolic blood pressure (mmHg)**	72.1 ± 8.8	75.5 ± 11.0	70.1 ± 11.3	< 0.001*	< 0.001*
**LDL-cholesterol (mmol/L)**	3.1 ± 0.9	2.6 ± 0.9	2.5 ± 1.0	< 0.001*	0.077
**HDL-cholesterol (mmol/L)**	1.4 ± 0.5	1.24 ± 0.44	1.17 ± 0.33	< 0.001*	< 0.001*
**Triglycerides (mmol/L)**	1.4 ± 1.0	1.6 ± 1.1	1.6 ± 1.2	< 0.001*	0.66
**Fasting glucose (mmol/L)**	6.8 ± 9.0	7.5 ± 6.3	8.5 ± 10.5	0.001*	0.037*
**Creatinine (μmol/L)**	79.8 ± 50.1	98.4 ± 74.6	136.6 ± 113.1	< 0.001*	< 0.001*
**Lipid-lowering therapy [*n*** **(%)]**	849 (58.2%)	2254 (70.8%)	452 (80.6%)	< 0.001*	< 0.001*
**25-hydroxyvitamin D (Ln)** ^‡^	3.02 ± 0.33	3.01 ± 0.33	2.98 ± 0.38	0.035*	0.043*
**Season of recruitment**				0.57	0.61
Spring [*n* (%)]	225 (15.4%)	515 (16.2%)	88 (15.7%)		
Summer [*n* (%)]	478 (32.7%)	940 (29.5%)	178 (31.7%)		
Autumn [*n* (%)]	222 (15.2%)	537 (16.9%)	99 (17.6%)		
Winter [*n* (%)]	535 (36.6%)	1193 (37.5%)	196 (34.9%)		
**Vitamin D GRS** ^⌞^	2.7 ± 1.2	2.7 ± 1.2	2.5 ± 1.3	0.001*	0.002*

*P < 0.05

^†^*P* value 1: Group (iii) versus (i)

*P* value 2: Group (iii) versus (ii)

^‡^Natural log-transformed due to skewed distribution

^⌞^GRS, genetic risk score (linear 0-6) based on allele scoring summation (CYP2R1: *rs2060793*; *GC: rs4588*, *rs7041*)

#### Hypertensive-diabetic subcohort sample

This sample comprised 3746 subjects who had prior history or diagnosis of hypertension per assessment at baseline (mean age: 65.8 ± 11.6 years, 62.7% male), with or without concomitant diabetes mellitus (type 2 diabetes: prevalence 78.9%). These subjects represented a sample of heightened underlying cardiovascular risk. The use of lipid-lowering therapy was up to 72.2%. A total of 17.5% of these subjects had a prior history of any of the following conditions: myocardial infarction, unstable angina, congestive heart failure, ischemic stroke, or peripheral vascular disease. They were prospectively followed up and studied for development of incident hard clinical CVD endpoints. The mean follow-up duration was 55.6 ± 28.9 months. The genetic instrumental variable of Vit-D prior obtained from the *Derivation Subcohort* was independently applied in this sample for causal inference of hard clinical CVD endpoints. To demonstrate consistency of findings, further sensitivity analyses were performed in which the primary analyses were repeated in 2954 subjects who were 100% co-morbid with hypertension and T2DM.

### Baseline demographic, clinical and laboratory assessments

Baseline demographic data, general cardiovascular risk factors, and definitions of clinical diagnoses were prior described [16]. Hypertension was defined as either resting systolic/diastolic blood pressure (BP) ≥ 140/90 mmHg at two different clinic visits or on medications. For outcome analyses, the JNC-8 guidelines were adopted in which the cut-offs for blood pressure control were systolic BP < 150 mmHg and diastolic BP < 90 mmHg (majority of subjects, 68%, were aged ≥ 60 years) [17]. The American Diabetes Association (ADA) guidelines for blood pressure control targets (systolic < 140 mmHg, diastolic < 90 mmHg) were adopted for subjects with 100% co-morbid hypertension and T2DM [7]. Diabetes mellitus was defined by serum fasting glucose ≥ 7.0 mmol/L or on medications. Fasting blood was collected for biochemical analysis of serum low-density/high-density lipoprotein (LDL/HDL)-cholesterol, triglycerides, glucose, and creatinine.

#### Laboratory measurements

Serum 25(OH)D concentration was measured using a validated enzyme immunoassay (Abbot, USA). We showed a small inter-assay variability between different validated enzyme immunoassays (correlation coefficient of 0.86, *P* < 0.001, using Abott, USA versus IDS Diagnostics, UK). We measured serum 25(OH)D in all subjects using the Abbot assay to enhance internal validity. For intra-assay variability, coefficient of variations for serum 25(OH)D ranged between 4.2 and 6.9% for the low 25(OH)D pool (mean 13.6 ng/mL [range 11.6–14.8]) and 1.9–2.5% for the high 25(OH)D pool (mean 36.8 ng/mL [range 35.6 to 38.4]) [18], with similar results shown in different populations [19]. Vit-D deficiency was defined by serum 25(OH)D < 20 ng/mL. Vit-D status was categorized as sufficient (≥ 30 ng/mL), insufficient (≥ 20 to < 30 ng/mL), or deficient (< 20 ng/mL). Seasonality of blood sampling was categorized [16].

### Derivation of genetically predicted vitamin D level

We considered candidate SNPs from various stages of Vit-D mechanistic pathways. Genotyping was performed using a specially designed high-throughput Exome chip array (Illumina HumanExome BeadChip, Asian exome-chip), as prior described [20, 21], to interrogate 12 single-nucleotide polymorphisms (SNPs) involved in the Vit-D mechanistic pathways and detected to have associations at genome-wide or subgenome-wide significance with serum 25(OH)D from prior genetic association or genome-wide associations studies (GWAS) (Biosynthetic: *rs4646536*, *rs10877012*, *rs3829251*, *rs1790349*; Activation: *rs2060793*, *rs1993116*; vitamin D-binding protein (VBP)/GC: *rs4588*, *rs7041*, *rs2282679*, *rs1155563*; and Vit-D receptor: *rs1544410*, *rs10735810*) [22–36]. Prior study supported that a simple unweighted score had similar utility compared to a more complex weighted score in Mendelian randomization analyses [37]. We constructed a simple point-scale 6-alleles genetic risk score (GRS, linear continuous: 0–6), with an approach similar to prior studies [38, 39], based on three SNPs that were significantly associated with serum 25(OH)D concentration in the *Derivation Subcohort*. Hardy-Weinberg equilibrium was tested by *X*^2^ against each SNP locus in the HKU-TRS cohort to exclude genotype-dependent ascertainment bias, using a prior validated equilibrium test calculator [40]. We further excluded linkage disequilibrium (pairwise) by testing against all SNP combinations included in the GRS. A constituent score of 0 to 2 of each SNPs was assigned to the total score according to the allele frequency distribution (AA, Aa, aa), thus deriving an overall score that ranged from 0 to 6. The scores of 0 and 6 respectively corresponded to the lowest and highest genetically predicted serum 25(OH)D concentration.

We obtained Mendelian randomization estimates for the associations of genetically predicted vitamin D status with comprehensive incident CVD events using two-sample instrumental variable analysis. Specifically, we obtained SNP-specific Wald’s estimates (quotient of genetic association on incident CVD events divided by genetic association on serological Vit-D) and then an overall estimate under an additive genetic model of the GRS (Wald’s estimate: βxy = βzy divided by βzx), and the ratio 95% confidence interval based upon Fieller’s theorem (E.C. Fieller, 1940) [41]. In brief, to meet the three basic assumptions in Mendelian randomization, i.e., including relevance, independence, as well as exclusions-restriction principle, we employed SNPs that strongly and independently predicted Vit-D levels. We sought to ascertain whether the genetic variants were independent of potential confounders from their crude associations with these variables in the HKU-TRS, including smoking history, use of lipid-lowering therapy, and BMI, and found that there was no significant association (all *P* > 0.20). To strengthen the assumption that such included SNPs were associated with incident CVD events only via serum 25(OH)D, we further checked for known direct effects of the genetic variants on CVD outcomes (i.e., horizontal pleiotropy, thus violating the exclusions-restriction principle) in three extensive, curated reference databases that recorded well-established associations between genotypes and phenotypes:

i. PhenoScanner (www.phenoscanner.medschl.cam.ac.uk);

ii. GWAS catalog (https://www.ebi.ac.uk/gwas/);

iii. Ensembl (http://www.ensembl.org/index.html);

A search through all these databases as of date of April 2021 found no relation of these SNPs of interests with potential confounders, including tobacco or alcohol use, physical activity, BMI, as well as socioeconomic position/Townsend index. As there was a limited number of SNPs included in our instrument, MR-Egger was not used for reasons that were prior explained [16].

### Comprehensive clinical CVD outcomes assessment

During the prospective follow-up, we adjudicated new onset CV diseases, and CV mortality based on the International Classification of Diseases, Ninth Revision (ICD-9), consisting of acute MI (ICD-9 410), unstable angina/acute coronary syndrome (ACS) (ICD-9 411.1), congestive heart failure (CHF) (ICD-9 428.0), ischemic stroke (ICD-9 433, 434, 435, 436), peripheral vascular disease (PVD) (ICD-9 443.9), and cardiovascular death (death certificate ICD-9 410-447). Data were retrieved and ascertained from the medical records and clinical data network of all public hospitals in Hong Kong, and from directly called back follow-ups.

The primary endpoint was the effect of genetically predicted Vit-D on combined CVD events, defined as occurrence of any of the following: incident MI, ACS, CHF, ischemic stroke, PVD, and CVD death. We defined CVD death as death directly resulting from circulatory disturbances as acute MI, acute/acute-on-chronic heart failure, cardiac arrhythmias, or ischemic/hemorrhagic stroke. Secondary endpoints were the effects of genetically predicted Vit-D on blood pressure and glycemic levels in hypertensive-diabetic subjects, and their impact on combined CVD events.

### Statistical analysis

Relations of serum 25(OH)D with binary clinical outcomes of CVD events and mortality were examined using logistic regression. Variables with skewed distribution were natural log-transformed during analysis. Serum 25(OH)D distribution had significant skewness and was natural log-transformed when appropriate. In the *Hypertensive-Diabetic Subcohort*, we used logistic and linear regression analyses to assess the relation of serum 25(OH)D with hypertension control, serum fasting glucose, lipid profile, and body-mass index, as appropriate. Cox proportional hazards regression was used to assess the relation of serum 25(OH)D with incident CVD events. Potential confounders were defined based on prior reported associations, specified a priori. Multivariable regression model was used to adjust for potential confounders including age, gender, smoking, diabetes mellitus, lipid-lowering therapy use, body-mass index, systolic/diastolic BP, creatinine, serum LDL/HDL cholesterol/ triglycerides, and seasonal variation of serum 25(OH)D measurement. To test for linearity assumptions, we repeated analyses with dichotomous models and categorical exposure variables when appropriate. Our study follow-up for primary endpoint had 100% completion. Any missing values were excluded from analyses.

A two-sided *P* value less than 0.05 was considered statistically significant. The statistical software packages IBM SPSS (version 21) (SPSS, Chicago, IL), STATA (version 14.0), GraphPad/ PRISM statistical calculators, and R-programming language (version 3.4.3) were used.

### Sensitivity analyses

As our study objective was to investigate the inferred effect of genetically predicted Vit-D on comprehensive clinical CVD events, analyses focused primarily on the constructed instrument GRS (CYP2R1: *rs2060793*; *GC: rs4588*, *rs7041*) and the primary endpoint. For sensitivity analyses of the genetic instrument, further testing using individual SNPs/their combinations were performed to demonstrate robustness and consistency. As the analyses were based on the constructed Vit-D GRS derived from a priori defined principles, there was no adjustment made for multiple comparisons.

Furthermore, Mendelian randomization outcome analyses in the hypertensive-diabetic subcohort were repeated among the group of subjects with strictly co-morbid hypertension and T2DM (*n* = 2954) (Supplementary Fig. 1).

## Results

### Serological 25(OH)D deficiency and constructed genetic instrument

The study flowchart is shown in Supplementary Fig. 1. Baseline clinical characteristics of the *Derivation Subcohort* and *Hypertensive-Diabetic Subcohort* are presented in Table 1, the latter stratified by the occurrence of incident combined CVD events. The prevalence of Vit-D deficiency in hypertensive-diabetic subjects (defined as serum 25(OH)D < 20 ng/mL) was 45.1%.

Based on the *Derivation Subcohort*, 6 SNPs from two genetic loci implicated in the Vit-D mechanistic pathways (CYP2R1: *rs2060793*, *rs1993116*; GC: *rs2282679*, *rs4588*, *rs1155563*, *rs7041*) were significantly associated with serum 25(OH)D concentration (all *P* < 0.05). We constructed a GRS based on 3 SNPs with the strongest prediction estimates for 25(OH)D without linkage disequilibrium (CYP2R1: *rs2060793*; GC: *rs4588*, *rs7041*). GRS was independently associated with serum 25(OH)D (B = 0.66 [95%CI 0.51 to 0.82], *P* < 0.001). Total variance of absolute serum 25(OH)D explained by GRS was 2.1%, with F-statistic = 32 (*P* < 0.001), indicating a strong instrument. Genetic variants included in our instrument showed inter-loci perfect equilibrium (*R*^2^ = 0%). *rs4588* and *rs7041* demonstrated only weak correlation (*R*^2^ = 0.16). Conveniently, per-allele increase in GRS predicted 15% reduced risk of Vit-D deficiency (OR = 0.85 [95%CI 0.81 to 0.89], *P* < 0.001, adjusted for age and sex). Tests for Hardy-Weinberg equilibrium revealed no violation (all SNPs *X*^2^ 0–1.5) in the HKU-TRS cohort.

Importantly, GRS was here independently derived from the above-mentioned analytic framework. We prior performed another two-sample Mendelian randomization analytic study based on the same HKU-TRS platform using a similar approach, but which specifically focused on a different theme of ischemic vascular events recurrence [16], and yielded a GRS comprising the same constituent SNPs. Such coherent findings substantiate the robustness of predictive utility of this SNPs combination as a validated GRS for serum 25(OH)D.

### Protection against combined CVD events in hypertensive-diabetic subjects

**Fig. 1:**
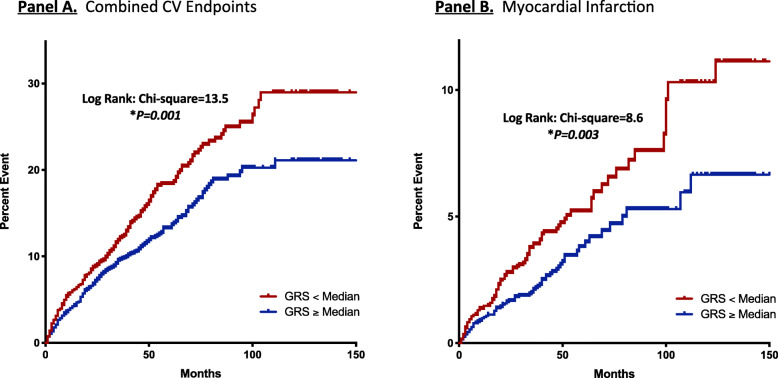
Cumulative hazards stratified by genetic vitamin D exposure for pre-specified clinical endpoints in hypertensive-diabetic subjects. Cumulative hazards curves supplemented with data on events-free survival. **A** Vitamin D genetic risk score (GRS) predicted combined cardiovascular (CV) endpoints in hypertensive subjects: mean survival (Below median: 123.6 [95%CI 119.8 to 127.5] months, versus ≥ median: 131.8 [95%CI 128.8 to 134.9] months, log-rank = 13.5, *P<0.001*); **B** Vitamin D GRS predicted incident myocardial infarction (MI): mean survival (below median: 145.1 [95%CI 142.6 to 147.7 months], versus ≥ median: 149.2 [95%CI 147.4 to 151.0 months], log-rank = 8.6, *P* = 0.003)

**Fig. 2:**
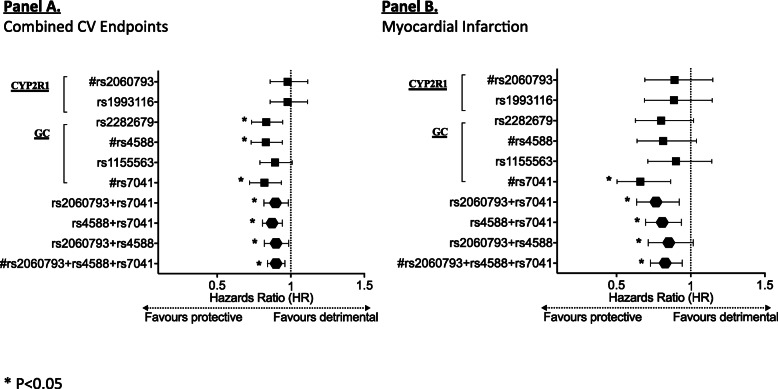
Per-allele estimates for hazards ratio (HR) of clinical endpoints driven by genetic vitamin D exposure in hypertensive-diabetic subjects. **A** Per-allele prediction estimates of candidate/constituent vitamin D genetic variants, or their combinations for combined cardiovascular (CV) endpoints are as follows: *rs2060793*: (HR = 0.98 [95%CI 0.86 to 1.12], *P* = 0.75); *rs1993116*: (HR = 0.98 [95%CI 0.86 to 1.11], *P* = 0.75); *rs2282679*: (HR = 0.83 [95%CI 0.73 to 0.95], *P* = 0.005); *rs4588*: (HR = 0.83 [95%CI 0.73 to 0.94], *P* = 0.004); *rs1155563*: (HR = 0.89 [95%CI 0.79 to 1.01], *P* = 0.070); *rs7041*: (HR = 0.82 [95%CI 0.72 to 0.94], *P* = 0.003); *rs4588*+*rs7041*: (HR = 0.87 [95%CI 0.81 to 0.94], *P* = 0.001); *rs2060793*+*rs7041*: (HR = 0.90 [95%CI 0.82 to 0.98], *P* = 0.020); *rs2060793*+*rs4588*: (HR= 0.90 [95%CI 0.82 to 0.99], *P=0.024*); *rs2060793*+*rs4588*+*rs7041*: (HR = 0.90 [95%CI 0.84 to 0.96], *P* = 0.002). The estimates were derived from multivariable Cox-proportional hazards model with adjustment for potential confounders as illustrated in Supplementary Table 1 (Multivariable Model 1).**B** Per-allele prediction estimates of candidate/constituent vitamin D genetic variants, or their combinations for incident myocardial infarction are as follows: *rs2060793*: (HR = 0.89 [95%CI 0.69 to 1.15], *P* = 0.38); *rs1993116*: (HR = 0.89 [95%CI 0.69 to 1.15], *P* = 0.36); *rs2282679*: (HR = 0.80 [95%CI 0.63 to 1.02], *P* = 0.07); *rs4588*: (HR = 0.82 [95%CI 0.64 to 1.04], *P* = 0.10); *rs1155563*: (HR = 0.90 [95%CI 0.71 to 1.14], *P* = 0.40); *rs7041*: (HR = 0.66 [95%CI 0.50 to 0.87], *P* = 0.003); *rs4588*+*rs7041*: (HR = 0.81 [95%CI 0.70 to 0.94], *P* = 0.005); *rs2060793*+*rs7041*: (HR = 0.77 [95%CI 0.64 to 0.92], *P* = 0.005); *rs2060793*+*rs4588*: (HR = 0.85 [95%CI 0.72 to 1.02], *P* = 0.08); *rs2060793*+*rs4588*+*rs7041*: (HR = 0.83 [95%CI 0.73 to 0.94], *P* = 0.004). The estimates were derived from multivariable Cox-proportional hazards model with adjustment for potential confounders (including age, diabetes mellitus, body-mass index, use of lipid-lowering drugs and creatinine), similar to multivariable model 1 illustrated in Supplementary Table 1

**Fig. 3:**
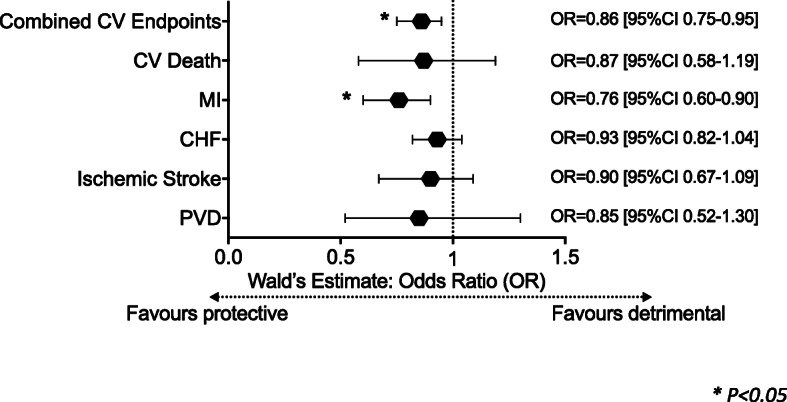
Mendelian randomization-inferred causality of vitamin D on incident clinical cardiovascular (CV) endpoints in hypertensive-diabetic subjects. Mendelian randomization showed vitamin D has causally protective effects against incident combined CV endpoints (Wald’s estimate: odds ratio [OR] = 0.86 [95%CI 0.75 to 0.95]) and incident myocardial infarction (OR = 0.76 [95%CI 0.60 to 0.90]) in 3746 hypertensive subjects. Any causality of vitamin D on incident CV death (OR = 0.87 [95%CI 0.58 to 1.19]), congestive heart failure (OR = 0.93 [95%CI 0.82 to 1.04]), ischemic stroke (OR = 0.90 [95%CI 0.67 to 1.09]), and peripheral vascular disease (OR = 0.85 [95%CI 0.52 to 1.30]) is neither supported nor excluded

After a mean follow-up duration of 55.6 ± 28.9 months, a total of 561 combined CVD events (15%) had occurred. Specifically, the number of incident events of MI, unstable angina, CHF, ischemic stroke, PVD, and CVD death were 162 (4.3%), 79 (2.1%), 372 (9.9%), 79 (2.1%), 22 (0.6%), and 35 (0.9%), respectively. Serological 25(OH)D deficiency (< 20 ng/mL) independently predicted incident combined CVD events (HR = 1.3 [95%CI 1.1 to 1.5], *P* = 0.007, Supplementary Table 2). Conversely, higher Vit-D GRS predicted a lower rate of incident combined CVD events in crude (HR 0.90 [95%CI 0.84 to 0.97], *P* = 0.003) and multivariable Cox regression analyses (HR 0.90 [95%CI 0.84 to 0.96], *P* = 0.002), Fig. 2). Kaplan-Meier analyses showed that lower GRS was associated with worsened event-free survival from combined CVD events (below median: 123.6 [95%CI 119.8 to 127.5] months, versus ≥ median: 131.8 [95%CI 128.8 to 134.9] months, log-rank = 13.5, *P* < 0.001, Fig. 1A). Applying two-sample Mendelian randomization, increased genetically predicted Vit-D is protective against combined CVD events in hypertensive subjects with prevailing T2DM (Wald’s estimate: OR = 0.86 [95%CI 0.75 to 0.95], Fig. 3). Sensitivity analyses including only subjects with strictly co-morbid hypertension and T2DM yielded similar findings (Wald’s estimate: OR = 0.87 [95%CI 0.76 to 0.96]).

### Protection against incident MI in hypertensive-diabetic subjects

Kaplan-Meier analyses revealed that lower Vit-D GRS was associated with reduced MI event-free survival (below median: 145.1 [95%CI 142.6 to 147.7 months], versus ≥ median: 149.2 [95%CI 147.4 to 151.0 months], log-rank = 8.6, *P* = 0.00*3*, Fig. 1B). Adjusted for potential confounders, per-allele increase in GRS remained independently predictive of reduced new-onset MI (HR = 0.83 [95%CI 0.73 to 0.94], *P* = 0.004, Fig. 2). Mendelian randomization indicates that increased genetically predicted Vit-D is protective against incident MI (Wald’s estimate: OR = 0.76 [95%CI 0.60 to 0.90]). Sensitivity analyses repeated among subjects with strictly co-morbid hypertension and T2DM yielded similar findings (Wald’ estimate OR = 0.75 [95%CI 0.58 to 0.89]).

As shown in Fig. 3, genetically predicted vitamin D status had no significant relations, albeit trends toward negative associations, with other secondary endpoints, including ischemic stroke, CHF, unstable angina, PVD, and CVD death alone in hypertensive-diabetic subjects. Of note, no relations were found between genetic Vit-D exposure and non-CVD death/all-cause mortality.

### Mechanistic role of blood pressure control in hypertension and T2DM

Relations of secondary endpoints, i.e., systolic/diastolic BP and fasting serum glucose, were examined against serological and genetically predicted 25(OH)D. Systolic BP, serum glucose, triglycerides, and LDL-cholesterol, as well as body-mass index had significant associations with serum 25(OH)D. However, systolic hypertension was the only dependent variable that was negatively predicted by increased GRS (Failed JNC-8 target of systolic BP < 150 mmHg: OR = 0.91 [0.86 to 0.97], *P* = 0.002, Supplementary Fig. 2A, Supplementary Table 2). Kaplan-Meier analyses in the *Hypertensive-Diabetic Subcohort* (4085 subjects, without or without genetic Vit-D measurements) showed that deviation from JNC-8 recommended cut-off for systolic hypertension control was associated with worsened combined CVD endpoints event-free survival (failed JNC-8: 120.0 [95%CI 114.1 to 126.0 months], versus adhered JNC-8: 131.6 [95%CI 129.2 to 134.1 months], log-rank = 16.0, *P* < 0.001, Supplementary Fig. 2B-i). Additionally, such deviation from JNC-8 recommended cut-off also predicted worsened incident MI-event free survival (failed JNC-8: 142.0 [95%CI 138.2 to 145.9 months] versus Adhered JNC-8: 148.7 [95%CI 147.1 to 150.2 months], log-rank = 20.5, *P* < 0.001, not shown in figures).

Mendelian randomization indicates that increased genetically predicted vitamin D status was protective against clinical deviation from achieving JNC-8 target for systolic hypertension control < 150 mmHg (Wald’s estimate: OR = 0.89 [95%CI 0.80 to 0.96]), as well as overall hypertension control (JNC-8: systolic BP < 150 mmHg and diastolic BP < 90 mmHg) (Wald’s estimate: OR = 0.92 [95%CI 0.84 to 0.98]).

### Impact of American Diabetes Association guideline-directed systolic hypertension control on CVD outcomes

Analyses repeated among strictly co-morbid hypertensive-diabetic subjects (*n* = 2954) showed that deviation from American Diabetes Association (ADA) guideline-directed systolic BP control (control target < 140 mmHg) predicted worsened combined CVD events-free survival (failed ADA: 124.2 [95%CI 119.5 to 129.0 months] versus adhered ADA: 131.6 [95%CI 128.3 to 135.0 months], log-rank = 11.5, *P* = 0.001). Similarly, deviation from ADA guideline-directed systolic BP control also predicted worsened incident MI-events free survival (failed ADA: 144.9 [95%CI 142.0 to 147.9 months] versus Adhered ADA: 147.9 [95%CI 145.7 to 150.2 months], log-rank = 11.7, *P* = 0.001, Supplementary Fig. 2B-ii).

### Preferrential protection in Vit-D deficient hypertensive-diabetic subjects

Each genetically instrumented rise of serum 25(OH)D by 1 ng/mL confers 14% reduced odds of combined CVD events in this group of hypertensive-diabetic subjects with prevailing Vit-D deficiency (45.1%), as well as 8% reduced odds of deviation from achieving JNC-8 hypertension control. Interestingly, subgroup analyses further revealed that the protective effect of increased vitamin D status against combined CVD events predominated among subjects with Vit-D deficiency (Wald’s estimate: OR = 0.85 [95%CI 0.71 to 0.96]), but was less apparent in those without Vit-D deficiency (OR = 0.90 [95%CI 0.77 to 1.03]). The protective effect of Vit-D against failed hypertension control also predominated among hypertensive subjects with Vit-D deficiency (Wald’s estimate: OR = 0.88 [95%CI 0.77 to 0.98], rather than in those without deficiency (OR = 0.91 [95%CI 0.80 to 1.01]).

## Discussion

To our knowledge, this is the first Mendelian randomization study that investigated vitamin D status for the focused secondary prevention of CVD events in high-risk persons with hypertension and T2DM.

Hypertension is two-fold as common in patients with T2DM, with prevalence estimates varying between 74 and 85% [8, 9]. Conversely, hypertensive subjects exhibit greater preponderance of insulin resistance than normotensive persons. The conjunctive presence of hypertension and T2DM exhibits synergistic effects in escalating CVD risks [10, 11]. Thus optimizing CVD risk control in this vulnerable group of hypertensive-diabetic subjects is of paramount importance in their clinical care. Vit-D deficiency is pandemic and highly reversible. Given its causal associations found with both T2DM [1–4] and hypertension [5, 6] in latest Mendelian randomization studies, it is imperative to know whether this further translates into clinically manifest CVD events and the underlying mechanisms.

Findings from our study indicated that a genetically predicted increase in serum 25(OH)D is protective against combined incident CVD events, incorporating CVD death, in these high-risk hypertensive-diabetic subjects. It is also protective against incident MI. Moreover, we further showed that genetically predicted Vit-D attenuates systolic hypertension and is a driving force for achieving guideline-directed blood pressure control in persons with T2DM and hypertension, resulting in reduced CVD events. Together with our recent Mendelian randomization focused analyses showing that genetically predicted increase in serum 25(OH)D protects against the clinical recurrence of ischemic stroke and myocardial infarction [16], the benefits of increased serum 25(OH)D appear to be conferred to high-risk subjects in the advanced spectrum of the cardiovascular continuum.

Our finding of genetically predicted higher serum 25(OH)D having ameliorating effects on systolic hypertension is consistent with prior literature. In a large meta-analyzed Mendelian randomization on hypertension, each 10% of genetically instrumented increase in serum 25(OH)D was causally associated with 8.1% decreased odds of hypertension [6]. Another large Danish prospective Mendelian randomization study subsequently showed similar findings [5]. Importantly, we made a solid step further and showed that better vitamin D status facilitated adherence to guideline-directed blood pressure control in hypertensive-diabetic subjects. This in turn translated into secondary reduction in CVD endpoints. Nevertheless, while prior Mendelian randomization studies supported a causal role of predicted decreases in serum 25(OH)D on the development of type 2 diabetes [3, 4], our data did not find a relation of genetically predicted Vitamin D status on fasting glucose level. The reason for this was unclear. The lack of current mechanistic exploration specifically on insulin resistance, a common condition linked to hypertension, is a limitation of our study. Prior Mendelian randomization studies on specific cardiovascular risk factors corroborate our findings of no significant effects of predicted changes in serum 25(OH)D on lipid profile [42].

The reason why predicted increases in serum 25(OH)D may lead to improved blood pressure control is at present unclear. Mechanistically, a lack of renin suppression that is associated with Vit-D deficiency may exacerbate hypertension. Other than facilitating hypertension control, there are several mechanisms through which Vit-D may confer secondary protection in high-risk hypertensive-diabetic subjects. Experimental studies showed that Vit-D promotes angiogenic myeloid cells homing to injured vasculature via stromal cell-derived factor (SDF1), enhancing re-endothelialization and vascular regeneration [43]. It also modulates activity of circulating endothelial progenitor cells [44] and macrophages [45], with a potential effect of retarding cellular senescence [46].

Our study has several important strengths. Firstly, given the effects of Vit-D on vascular regeneration and repair [43], our study hypothesis specifically focused on the secondary prevention of high-risk subjects with hypertension and T2DM. This area of secondary protection has been largely unexplored by prior studies. Secondly, this is a prospective Mendelian randomization study with, to our knowledge, the most comprehensive interrogation of incident CVD hard clinical endpoints, incorporating CVD death. Thirdly, our high-throughput exome-chip screened 12 candidate SNPs from comprehensive Vit-D mechanistic pathways and derived a GRS with R^2^ of 2.1% and F-statistic of 32, which has a good explanatory effect size for serum 25(OH)D among Mendelian randomization studies focusing on cardiac outcomes with equivalent sample size or above [38, 39, 47]. Despite large sample sizes, genetic instruments used in the prior several Mendelian randomization studies had typically R^2^ for variance of serum 25(OH)D less than, or at best equal to 1.0% [38, 39]. Furthermore, we used individual data rather than pooled summary statistics. Prior GWAS revealed that variance of predicted changes in serum 25(OH)D was explained by genetic variants of Vit-D pathways by no more than 5% [30]. It is known that Mendelian randomization analyses using two-sample summary statistics are preferentially biased toward null especially in the case of a weak instrument [48], which could be exactly the case with Vit-D. Fourthly, our prospective follow-up rate of 100% with high incident event rates (15% primary endpoints over 5 years) maximized the cohort’s ability to detect any differences in CVD outcomes in hypertensive-diabetic subjects. Fifthly, the 45% prevalence of Vit-D deficiency in our hypertensive-diabetic sample provided excellent variability in serological Vit-D status for the study of causal inference.

Nonetheless, the choice of SNPs included in this investigation was limited by literature available at the time of study conception. Subsequent studies had identified further important genetic variants [49], constituting a limitation of our study. While acknowledging that a possibility of residual biases or confounding in either directions, or presence of horizontal pleiotropy cannot be completely excluded, we carefully studied each included SNP (*rs2060793*, *rs4588*, *rs7041*) in three extensive reference genetic databases. We found that none of the genetic variants included was related to key potential confounders such as tobacco or alcohol use, physical activity, BMI, as well as socioeconomic position.

## Conclusions

We conclude that increased genetically predicted serum 25(OH)D has a secondary protective effect against incident combined CVD events and MI in high-risk Chinese subjects with hypertension and T2DM. Such effects are mediated at least partially through ameliorated hypertension, and predominantly benefit those with serologically 25(OH)D deficiency (Supplementary Figure 3).

## Supplementary Information

**Additional file 1: Supplementary Figure 1.** Study Flowchart. Study flowchart detailing the HKU-TRS Cohort, with the methodological framework of *Derivation Subcohort* (*n*=1460) and the *Hypertensive-Diabetic Subcohort* (*n*=3746) samples that underwent two-sample Mendelian randomization for causal inference of Vitamin D on prospective cardiovascular endpoints in hypertensive subjects with prevailing type 2 diabetes. Supplementary Figure 2. *Panel A.* Serological and Genetic Vitamin D Prediction Risk Estimates for Deviation from Achieving Guideline-Directed Systolic Hypertension Control. Adjusted for sex and age, per-standard deviation increase in serum 25-hydroxyvitamin D and per-allele increase in Genetic Risk Score (GRS) respectively predicted 11% (OR=0.89 [95%CI 0.83 to 0.96], *P=0.002*) and 9% (OR=0.91 [0.86 to 0.97], *P=0.002*) reduced odds of deviation from JNC-8 guideline-recommended target for systolic hypertension (systolic <150mmHg). *(PDF 398 kb) Panel B.* (i). Failed JNC-8 Guideline Cut-off For Systolic Hypertension Predicts Risk of Combined Cardiovascular (CV) Events. Kaplan-Meier analysis showing deviation from JNC-8 recommended treatment target for systolic hypertension (<150mmHg) was associated with worsened combined CV endpoints event-free survival (120.0 [95%CI 114.1 to 126.0 months] versus 131.6 [95%CI 129.2 to 134.1 months], log-rank=16.0, *P<0.001*); (ii). Failed ADA Guideline Cut-off For Systolic Hypertension Predicts Risk of Combined CV Events. Deviation from ADA guideline-directed systolic BP control (control target <140mmHg) predicted worsened combined CVD events-free survival (124.2 [95%CI 119.5 to 129.0 months] versus 131.6 [95%CI 128.3 to 135.0 months], log-rank=11.5, *P=0.001*). Supplementary Figure 3. Theoretical Paradigm. Two-sample Mendelian randomization supports causality of Vitamin D protecting against incident combined CVD events and myocardial infarction in hypertensive-diabetic subjects, at least partially through facilitating guideline-directed clinical control of blood pressure.

**Additional file 2: Supplementary Table 1.** Serological and Genetic Vitamin D Exposure in Prediction Models for Combined Cardiovascular (CV) Endpoints in Hypertensive-Diabetic Subjects ^†^.

**Additional file 3: Supplementary Table 2.** Prediction estimates of Vit-D GRS^⌞^ for cardiovascular (CV) Risk Factors ^†^.

## Data Availability

Data generated during this study will not be made publicly available, because the act of data sharing had not been incorporated as part of the original study protocol at the time of Ethics Committee review and approval. The authors have given the best efforts in presenting the comprehensive and relevant data in the current manuscript.
