# Supplementary material for: Mendelian randomization analysis of vitamin D in the secondary prevention of hypertensive-diabetic subjects: role of facilitating blood pressure control
Source: Genes Nutr. 2022 Jan 29;17:1. doi: 10.1186/s12263-022-00704-z (PMC8903706; doi:10.1186/s12263-022-00704-z)

Figure 1.
Kaplan-Meier Curves Analyses for Clinical Cardiovascular Endpoints Stratified by Vit-D Genetic Exposure

Panel A. Combined CV Endpoints

Panel B. Myocardial Infarction

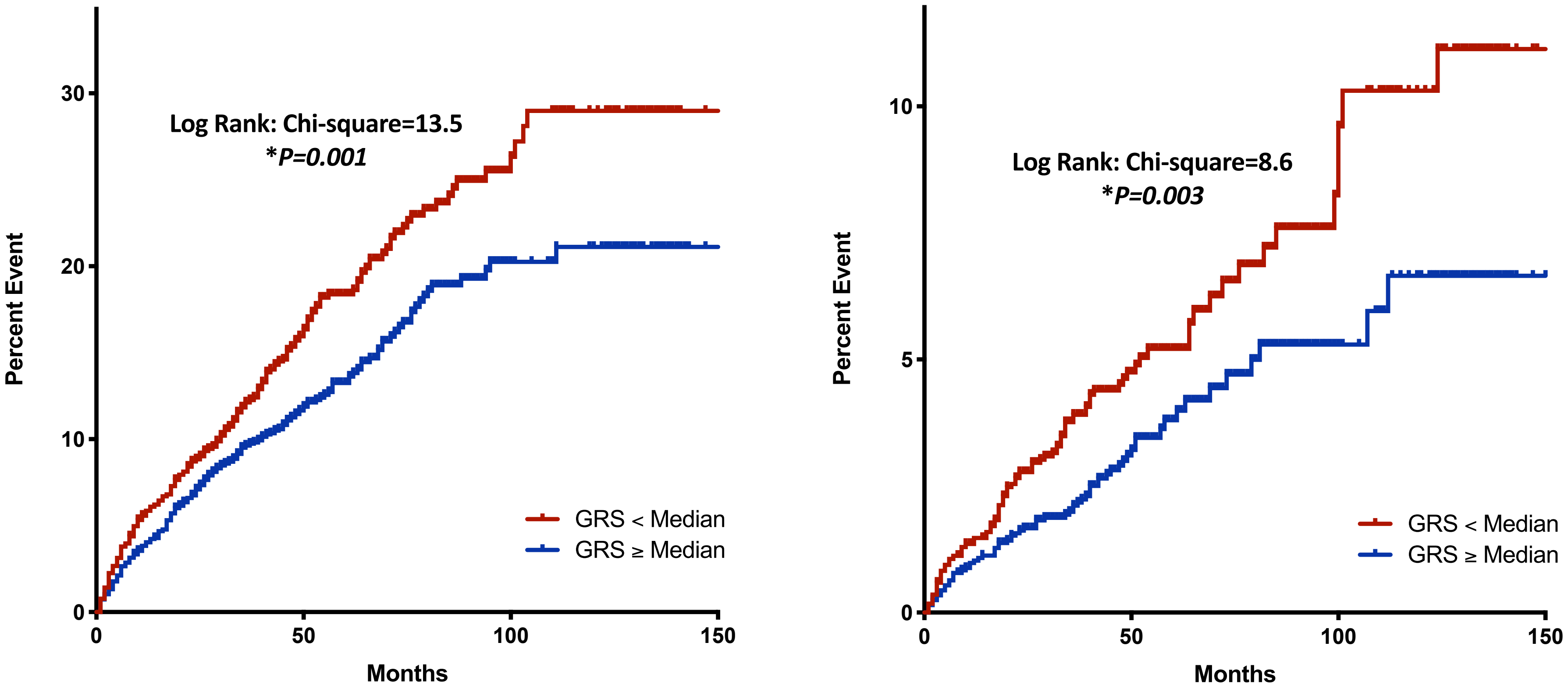

Figure 2. **Per-Allele Estimates of Hazards Ratio (HR) for Incident Clinical CV Endpoints Driven by Genetic Vit-D Exposure**

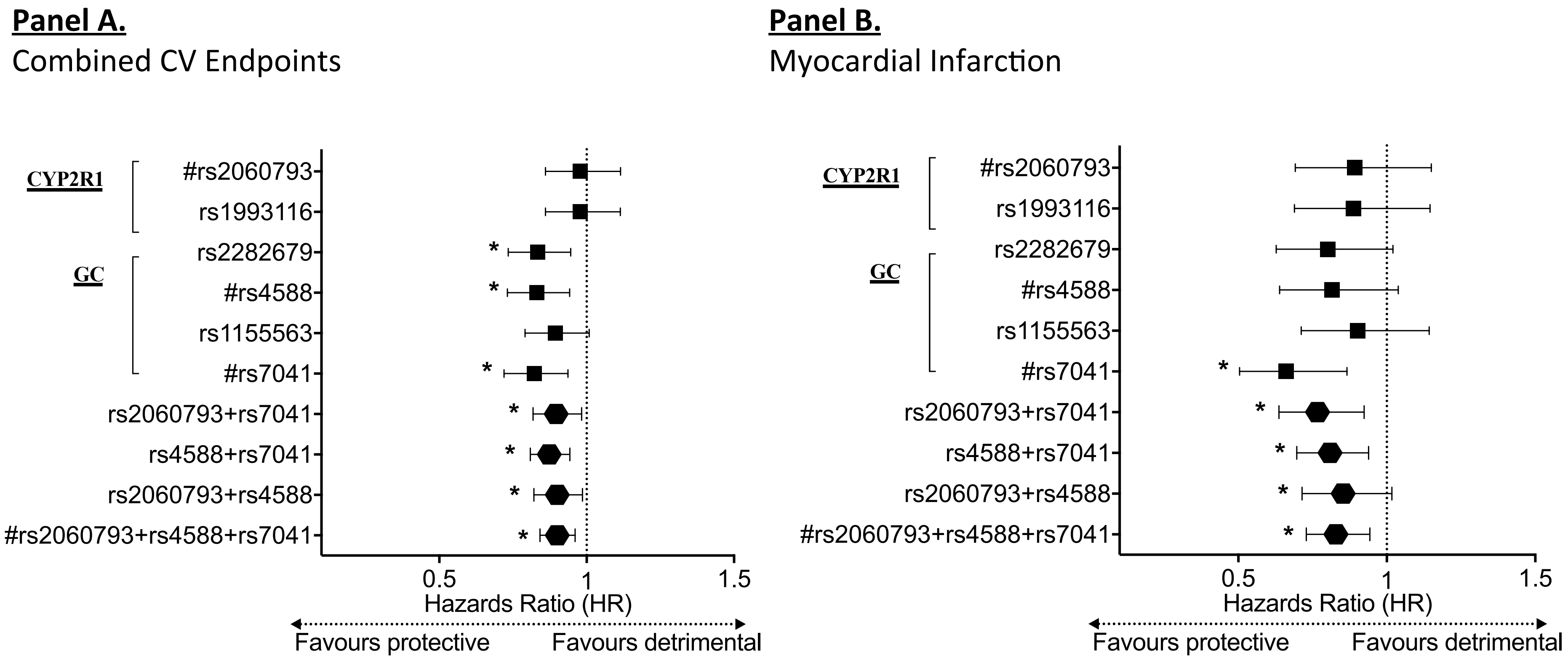

* P<0.05

Figure 3.

Mendelian Randomization-Inferred Causality of Vit-D on Incident Clinical CV Endpoints

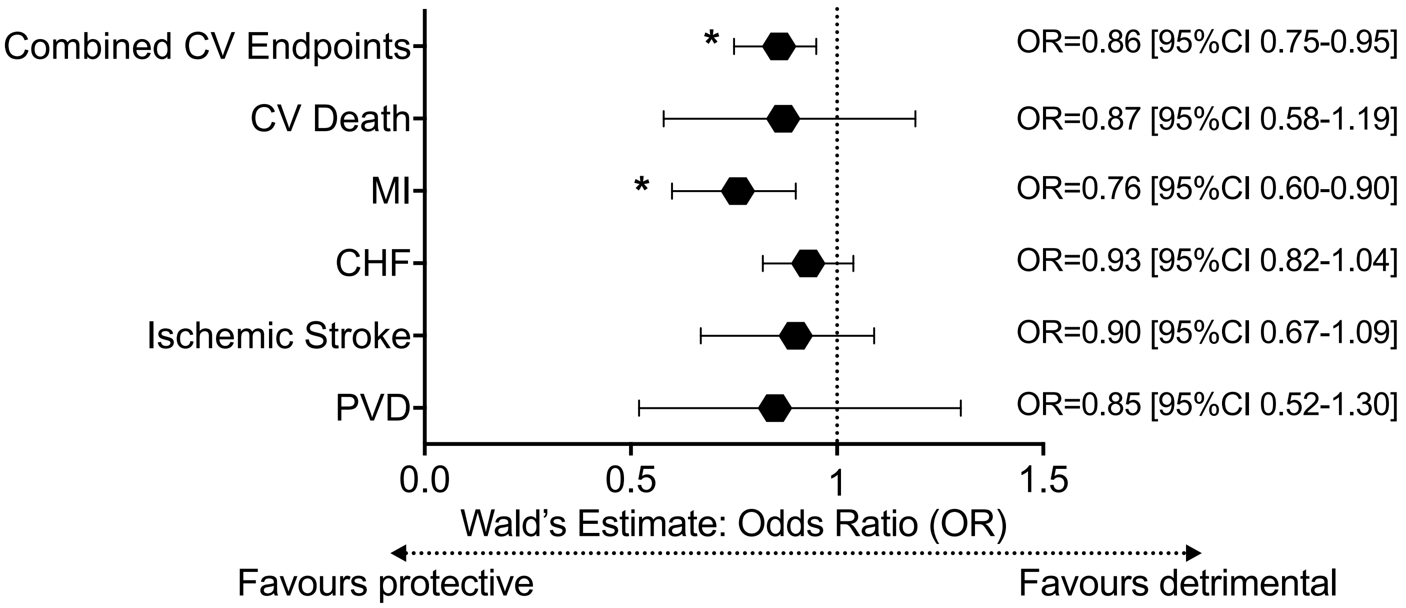

** P<0.05*

Supplementary Figure 1

STUDY FLOWCHART

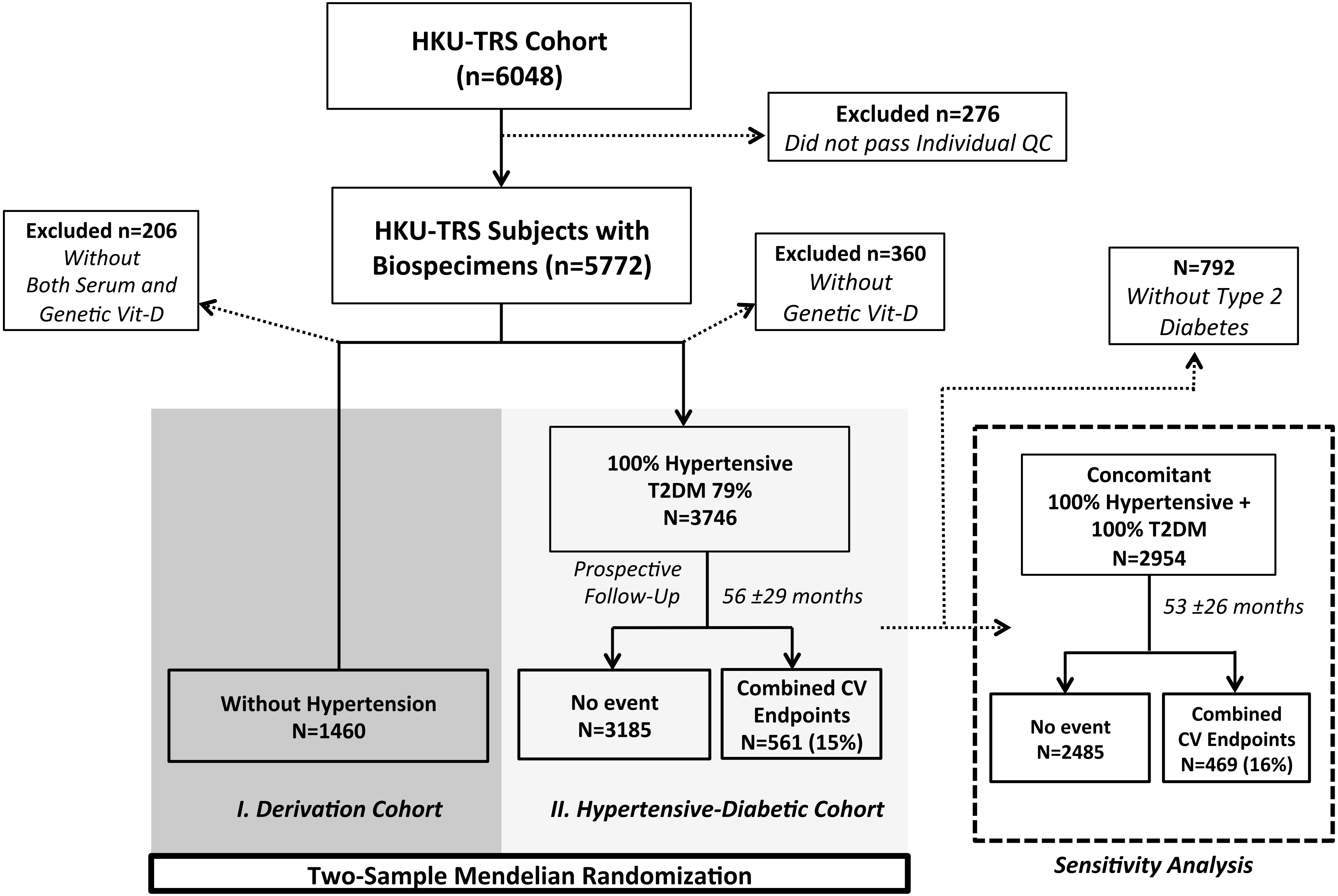

Supplementary Figure 2.

Panel A. Serological and Genetic Vitamin D Prediction Risk Estimates for Deviation from Achieving Guideline-Directed Systolic Hypertension Control

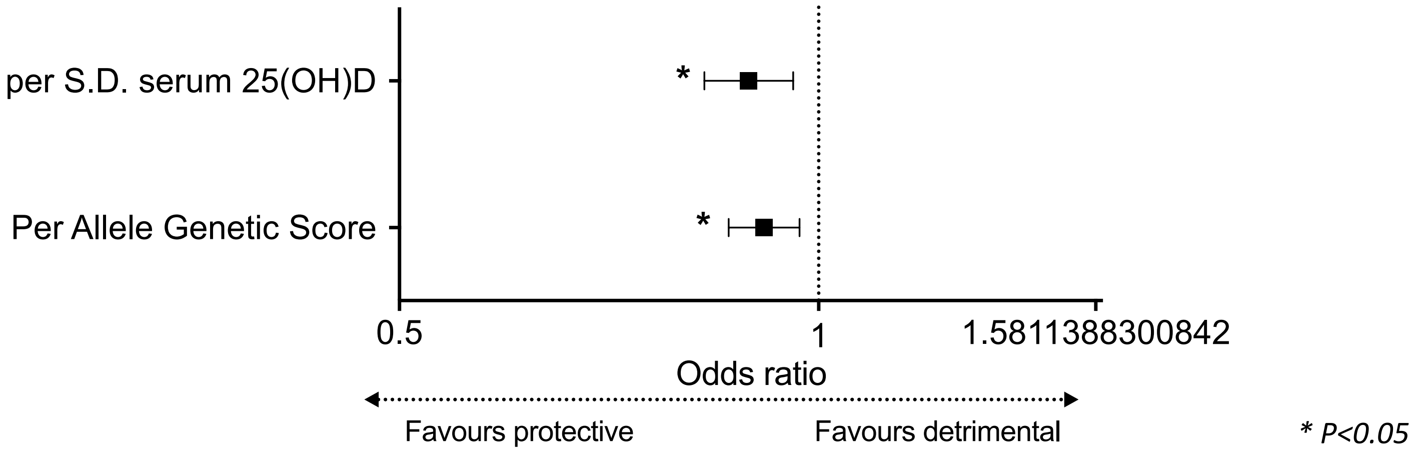

Panel B. Failed (i) JNC-8 and (ii) ADA Guidelines-Directed Cut-off For Systolic Hypertension Predicts Risk of Incident Combined CV Endpoints.

(i).

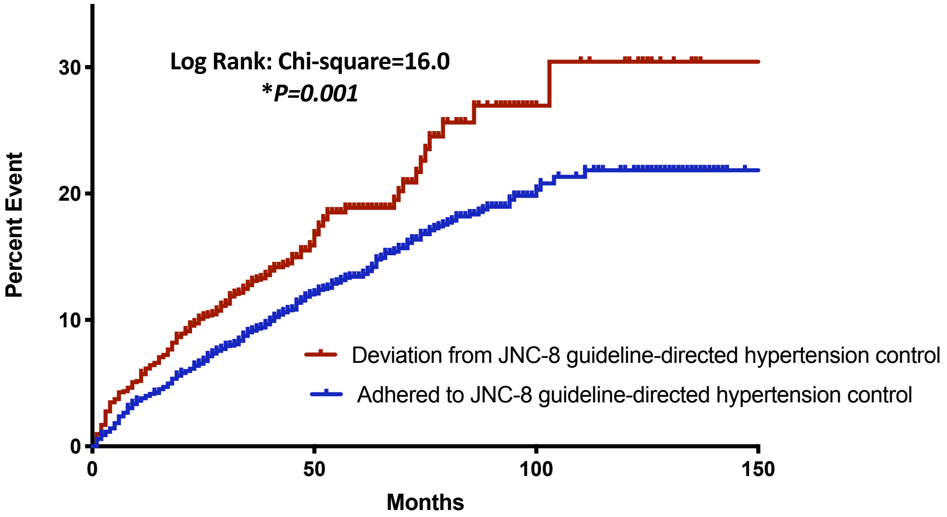

(ii).

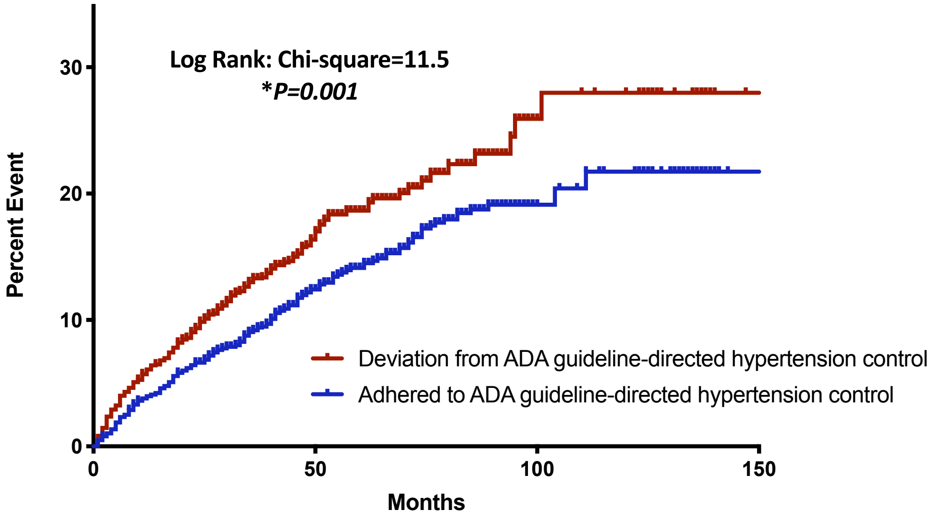

Supplementary Figure 3.

Theoretical Paradigm

Genetically Predicted Vit-D Confers Secondary Protection Against CVD Events in Hypertensive-Diabetic Subjects via Facilitating Blood Pressure Control

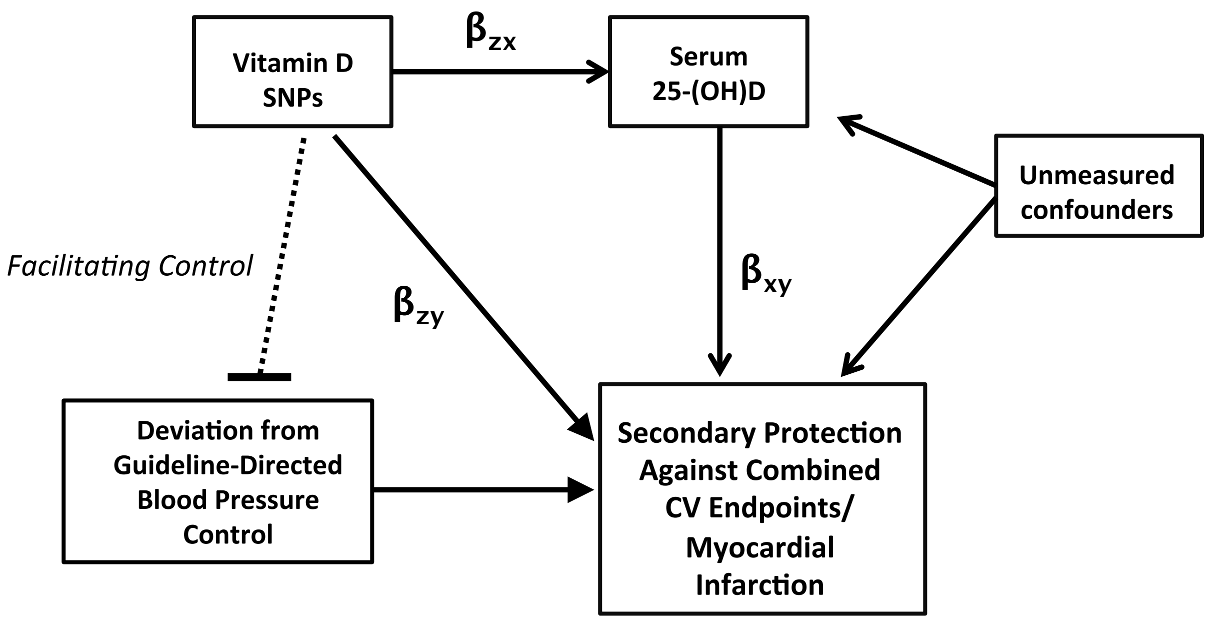

Supplement: Supplementary file 1 — Additional file 1: Supplementary Figure 1. Study Flowchart. Study flowchart detailing the HKU-TRS Cohort, with the methodological framework of Derivation Subcohort (n=1460) and the Hypertensive-Diabetic Subcohort (n=3746) samples that underwent two-sample Mendelian randomization for causal inference of Vitamin D on prospective cardiovascular endpoints in hypertensive subjects with prevailing type 2 diabetes. Supplementary Figure 2. Panel A. Serological and Genetic Vitamin D Prediction Risk Estimates for Deviation from Achieving Guideline-Directed Systolic Hypertension Control. Adjusted for sex and age, per-standard deviation increase in serum 25-hydroxyvitamin D and per-allele increase in Genetic Risk Score (GRS) respectively predicted 11% (OR=0.89 [95%CI 0.83 to 0.96], P=0.002) and 9% (OR=0.91 [0.86 to 0.97], P=0.002) reduced odds of deviation from JNC-8 guideline-recommended target for systolic hypertension (systolic <150mmHg). (PDF 398 kb) Panel B. (i). Failed JNC-8 Guideline Cut-off For Systolic Hypertension Predicts Risk of Combined Cardiovascular (CV) Events. Kaplan-Meier analysis showing deviation from JNC-8 recommended treatment target for systolic hypertension (<150mmHg) was associated with worsened combined CV endpoints event-free survival (120.0 [95%CI 114.1 to 126.0 months] versus 131.6 [95%CI 129.2 to 134.1 months], log-rank=16.0, P<0.001); (ii). Failed ADA Guideline Cut-off For Systolic Hypertension Predicts Risk of Combined CV Events. Deviation from ADA guideline-directed systolic BP control (control target <140mmHg) predicted worsened combined CVD events-free survival (124.2 [95%CI 119.5 to 129.0 months] versus 131.6 [95%CI 128.3 to 135.0 months], log-rank=11.5, P=0.001). Supplementary Figure 3. Theoretical Paradigm. Two-sample Mendelian randomization supports causality of Vitamin D protecting against incident combined CVD events and myocardial infarction in hypertensive-diabetic subjects, at least partially through facilitating guideline-directed cl [file 12263_2022_704_MOESM1_ESM.pdf]
