# Supplementary material for: Mendelian randomization analysis of vitamin D in the secondary prevention of hypertensive-diabetic subjects: role of facilitating blood pressure control
Source: Genes Nutr. 2022 Jan 29;17:1. doi: 10.1186/s12263-022-00704-z (PMC8903706; doi:10.1186/s12263-022-00704-z)
Supplement: Supplementary file 2 — Additional file 2: Supplementary Table 1. Serological and Genetic Vitamin D Exposure in Prediction Models for Combined Cardiovascular (CV) Endpoints in Hypertensive-Diabetic Subjects †. [file 12263_2022_704_MOESM2_ESM.docx]

| **SUPPLEMENTARY TABLE 1.**  Serological and Genetic Vitamin D Exposure in Prediction Models for Combined Cardiovascular (CV) Endpoints in Hypertensive-Diabetic Subjects ^†^. | | | | | | |
| --- | --- | --- | --- | --- | --- | --- |
|  | **Crude Model** ^‡^ |  | **Multivariable Model 1** ^§^ |  | **Multivariable Model 2** ^#^ |  |
|  | HR [95%CI] *^2^* | P-value | HR [95%CI] *^2^* | P-value | HR [95%CI] *^2^* | P-value |
| **Age (years)** | 1.06 [1.055 to 1.072] | *<0.001** | 1.06 [1.05 to 1.07] | *<0.001** | 1.07 [1.06 to 1.08] | *<0.001** |
| **Male** | 0.97 [0.82 to 1.15] | 0.97 | - | - | 0.95 [0.77 to 1.17] | 0.60 |
| **Smoking** | 1.23 [1.05 to 1.45] | *0.013** | 1.17 [0.99 to 1.39] | 0.067 | 1.19 [0.97 to 1.46] | 0.10 |
| **Body-mass index (kg/m^-2^)** | 1.004 [0.98 to 1.03] | 0.68 | - | - | 1.05 [1.02 to 1.07] | *<0.001** |
| **Diabetes mellitus** | 1.47 [1.18 to 1.82] | *0.001** | 1.69 [1.35 to 2.12] | *<0.001** | 1.61 [1.25 to 2.07] | *<0.001** |
| **Lipid-Lowering Therapy** | 1.80 [1.47 to 2.19] | *<0.001** | 1.28 [1.03 to 1.58] | *0.026** | 1.26 [1.01 to 1.58] | *0.037** |
| **Serum Creatinine (µmol/L)** | 1.002 [1.002 to 1.003] | *<0.001** | 1.002 [1.002 to 1.003] | *<0.001** | 1.003 [1.002 to 1.003] | *<0.001** |
| **Recruitment Season** Spring | Ref |  |  |  |  |  |
| Summer | 1.17 [0.91 to 1.49] | 0.23 | - | - | 1.17 [0.89 to 1.53] | 0.26 |
| Autumn | 1.12 [0.85 to 1.49] | 0.41 | - | - | 1.19 [0.88 to 1.61] | 0.25 |
| Winter | 1.04 [0.82 to 1.33] | 0.75 | - | - | 1.04 [0.80 to 1.36] | 0.76 |
| **Serological Vitamin D Deficiency**  25-hydroxyvitamin D <20ng/mL | 1.25 [1.06 to 1.46] | *0.007** | 1.25 [1.06 to 1.48] | *0.007** | 1.21 [1.02 to 1.44] | *0.029** |
| **Genetic Vitamin D Exposure**  Vitamin D GRS | 0.90 [0.84 to 0.97] | *0.003** | 0.90 [0.84 to 0.96] | *0.002** | 0.91 [0.85 to 0.98] | *0.008** |

*** statistically significant at 95% confidence (*P<0.05*)

^†^ Hazard ratio (HR) prediction estimates and 95% confidence interval for combined CV endpoints explained by variable of interest as shown by univariable and multivariable Cox proportional hazards regression; ^‡^ Crude Model: unadjusted; ^§^ Multivariable Model 1: adjusted for potential confounders with P-value ≤0.10 in Crude Model; ^#^ Multivariable Model 2: adjusted for all potential confounders as defined *a priori;*  GRS, Genetic Risk Score (linear 0-6) based on combined allele scoring summation (CYP2R1: *rs2060793*; GC: *rs4588*, *rs7041*);
