# Supplementary material for: Mendelian randomization analysis of vitamin D in the secondary prevention of hypertensive-diabetic subjects: role of facilitating blood pressure control
Source: Genes Nutr. 2022 Jan 29;17:1. doi: 10.1186/s12263-022-00704-z (PMC8903706; doi:10.1186/s12263-022-00704-z)
Supplement: Supplementary file 3 — Additional file 3: Supplementary Table 2. Prediction estimates of Vit-D GRS⌞ for cardiovascular (CV) Risk Factors †. [file 12263_2022_704_MOESM3_ESM.docx]

**SUPPLEMENTARY TABLE 2.** Prediction estimates of Vit-D GRS for cardiovascular (CV) Risk Factors ^†^.

| *Primary Analyses*  **Hypertensive-Diabetic**  **Subcohort (n=3746)** | Regression Estimate [95%CI] ^†^ | P-value | *Sensitivity Analyses*  **Co-Morbid Hypertensive-Diabetic**  **Subjects (n=2594)** | Regression Estimate [95%CI] ^†^ | P-value |
| --- | --- | --- | --- | --- | --- |
| **Hypertension Control** |  |  | **Hypertension Control** |  |  |
| Systolic BP | B=-0.48 [-0.97 to 0.02] | 0.061 | Systolic BP | B=-0.62 [-1.19 to -0.06] | *0.031** |
| Diastolic BP | B=-0.14 [-0.40 to 0.12] | 0.30 | Diastolic BP | B=-0.21 [-0.50 to 0.07] | 0.15 |
| Failed JNC-8 Systolic BP <150mmHg | OR=0.91 [0.86 to 0.97] | *0.002** | Failed JNC-8 Systolic BP <150mmHg | OR=0.88 [0.83 to 0.94] | *<0.001** |
| Failed JNC-8 diastolic BP <90mmHg | OR=0.97 [0.89 to 1.06] | 0.51 | Failed ADA Systolic BP <140mmHg | OR=0.95 [0.89 to 1.01] | 0.080 |
|  |  |  | Failed ADA/ JNC-8 diastolic BP  <90mmHg | OR=0.96 [0.87 to 1.07] | 0.45 |
| **Glycemic Control** |  |  | **Glycemic Control** |  |  |
| Fasting glucose (mmol/L) | B=-0.02 [-0.21 to 0.16] | 0.81 | Fasting glucose (mmol/L) | B=0.02 [-0.13 to 0.16] | 0.82 |
| **Lipid Profile** |  |  | **Lipid Profile** |  |  |
| LDL-c (mmol/L) | B=0.02 [-0.01 to 0.04] | 0.16 | LDL-c (mmol/L) | B=0.02 [-0.004 to 0.05] | 0.10 |
| HDL-c (mmol/L) | B=0.003 [-0.01 to 0.02] | 0.54 | HDL-c (mmol/L) | B=0.001 [-0.01 to 0.01] | 0.82 |
| Triglycerides (mmol/L) | B=0.01 [-0.02 to 0.04] | 0.54 | Triglycerides (mmol/L) | B=0.02 [-0.02 to 0.05] | 0.35 |
| **Obesity Risk** |  |  | **Obesity Risk** |  |  |
| Body mass index (kgm^-2^) | B=-0.02 [-0.13 to 0.08] | 0.66 | Body mass index (kgm^-2^) | B=-0.02 [-0.14 to 0.10] | 0.79 |

GRS, Genetic Risk Score comprising *rs2060793*, *rs4588* and *rs7041*

^†^ Multivariable logistic or linear regression as appropriate, adjusted for sex and age

* *P<0.05*
